# When Legacy Triumphs: Resolving Leadless Pacemaker Programming Failure Caused by Loop Recorder Interference

**DOI:** 10.1111/pace.70055

**Published:** 2025-09-17

**Authors:** Gabriele Pavani, Antonino Previti, Paolo Garrone, Gianpaolo Varalda, Emma Fratini, Alfonso Franzè, Alessandra Chinaglia

**Affiliations:** ^1^ Cardiac Electrophysiology Unit San Luigi Gonzaga University Hospital, Orbassano Turin Italy; ^2^ Interventional Cardiology Unit San Luigi Gonzaga University Hospital, Orbassano Turin Italy; ^3^ University of Turin Turin Italy

**Keywords:** Biotronik BIOMONITOR, device interaction, electromagnetic compatibility (EMC), electromagnetic interference (EMI), implantable loop recorder (ILR), leadless pacemaker, Medtronic Carelink 2090, Medtronic Micra, Medtronic SmartSync, programmer communication

## Abstract

The growing prevalence of co‐implanted cardiac electronic devices increases the potential for clinically significant interactions. We describe the case of an 81‐year‐old man with a Biotronik BIOMONITOR IV implantable loop recorder (ILR) who required a Medtronic MICRA VR leadless pacemaker. Post‐implantation, interrogation with the modern Medtronic SmartSync programmer failed due to suspected electromagnetic interference from the ILR. Communication was successfully established using the older Medtronic CareLink 2090 programmer. This report highlights a unique instance of inter‐device interference, underscoring the importance of understanding programmer technology and device compatibility in complex clinical scenarios.

## Graphical abstract

Interference from a Biotronik loop recorder while programming a Medtronic MICRA pacemaker. A graphical abstract illustrating the failure of the Medtronic SmartSync programmer (operating at 175 kHz via near‐field inductive coupling) and the success of the older Medtronic VITATRON 2090 programmer (operating at ∼400 MHz via far‐field RF telemetry) to connect with the MICRA leadless pacemaker in the presence of an implanted Biotronik loop recorder.

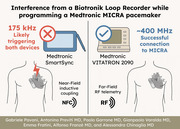

**Abbreviations**
CIEDs: cardiac implantable electronic devices
EMI: electromagnetic interference
GHz: gigahertz
ICD: implantable cardioverter‐defibrillator
ILR: implantable loop recorder
kHz: kilohertz
MHz: megahertz
RF: radio frequency
VAD: ventricular assist device

## 1 Introduction

The fields of cardiac electrophysiology have been significantly advanced by the development of compact, leadless pacemakers and long‐term implantable loop recorders (ILRs). While these technologies offer less invasive approaches to monitoring and therapy, the concurrent use of devices from different manufacturers introduces new challenges in clinical management. Electromagnetic interference (EMI) represents a known risk for cardiac implantable electronic devices (CIEDs), with sources varying from external fields to interactions between adjacent devices (Tables 1).

**TABLE 1:** Relevant cases of programmer/CIED interference.

Citation	Summary	Devices involved	Programmer/Activator
Duru, et al. [1]	Hospital pager interfered with pacemaker telemetry due to 36 kHz overlap	Various pacemakers	Pacemaker programmers (32–37 kHz)
Thaker et al. [2]	iPod interfered with Medtronic Reveal ILR telemetry	Medtronic reveal plus ILR	Medtronic ILR programmer + activator
Wang et al. [3]	Abandoned pacemaker caused inhibition in leadless pacemaker	Unknown leadless + old pacemaker	Leadless programmer
Tomomori et al. [4]	Impella VAD blocked ICD telemetry	Biotronik ICD + Impella	Biotronik programmer
Wu et al. [5]	Smartphone disrupted telemetry in pacemakers	Various CIEDs	Multiple programmers
Pinski and Trohman [6]	General EMI sources affecting pacemakers	Pacemakers/ICDs	Review
Hayes et al. [7]	Cellphones caused asynchronous pacing	Pacemakers	N/A

Abbreviations: CIEDs, cardiac implantable electronic devices; ICD, implantable cardioverter‐defibrillator; ILR, implantable loop recorder; PACE, *Pacing and Clinical Electrophysiology*; VAD, ventricular assist device.

This report details a unique clinical challenge where suspected EMI originating from a Biotronik ILR disabled the telemetry of a contemporary programmer attempting to communicate with a Medtronic MICRA pacemaker, whereas a legacy system functioned perfectly. This event prompts a critical evaluation of device compatibility, telemetry architecture, and system design. Here, we analyze the technical basis of this interaction, review similar occurrences, and propose practical considerations for clinicians managing patients with multiple CIEDs, reinforcing the value of older technologies in resolving modern clinical problems.

## 2 Case Presentation

Written informed consent was obtained from the patient for the publication of this case report and any accompanying images.

We present the case of an 81‐year‐old gentleman with a multifaceted medical background, including chronic lymphocytic leukemia managed with acalabrutinib and a history of prostate adenocarcinoma treated successfully with multimodal therapy. His history was also notable for Parkinson's disease with dysphagia and a significant frailty profile.

His cardiovascular history included permanent atrial fibrillation, managed with rate control and anticoagulation, and a prior surgical repair for an ascending aortic dissection. In early 2025, he presented with recurrent syncopal episodes. An implanted Biotronik Biomonitor IV ILR subsequently documented multiple symptomatic bradyarrhythmic episodes with pauses up to eight seconds, establishing an indication for permanent pacing.

**FIGURE 1:**
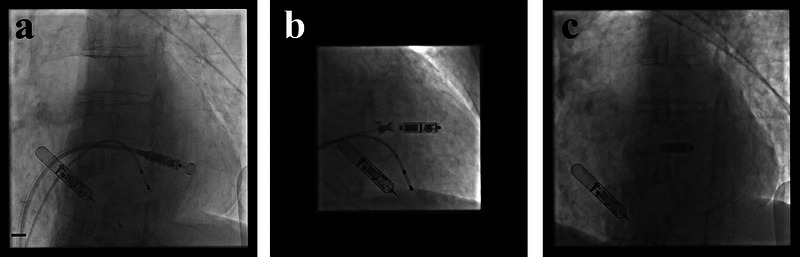
Intraoperative imaging of MICRA implantation. (a) Initial fluoroscopic view showing the catheter delivery system with the MICRA and the proximity of the ILR. (b) Fluoroscopic deployment view of the MICRA pacemaker in the mid‐septal right ventricular wall. (c) Post‐deployment positioning check showing both the MICRA leadless pacemaker and the ILR in situ.

Given his complex history, which elevated the risk for infection and complicated vascular access, a leadless pacing strategy was chosen. In April 2025, a Medtronic MICRA MC1VR01 was implanted without procedural complications (Figure 1). However, immediate post‐implant device interrogation with the Medtronic CareLink SmartSync programmer failed, presumably due to interference from the nearby ILR. The programmer's screen did not display any specific error message, simply failing to establish and maintain a stable telemetry session. Communication was successfully established using a previous‐generation Medtronic CareLink 2090 programmer, which was possible because the implanted device was an older MICRA model. Initial electrical parameters were satisfactory.

At the one‐month follow‐up, we investigated the interference further. We found that by actively interrogating the ILR with its dedicated Biotronik programmer, we could concurrently establish a stable telemetry session with the MICRA using the SmartSync programmer, particularly when its wand was positioned more laterally on the chest. This suggested that the combination of wand repositioning and active engagement of the ILR mitigated the interference.

## 3 Discussion

This case highlights the necessity of a sophisticated understanding of CIED telemetry systems, especially when managing co‐implanted devices. The interference likely originated from a combination of technical factors. The SmartSync programmer's 175 kHz near‐field inductive telemetry is vulnerable to low‐frequency noise, which could have been emitted by the ILR's internal circuits. In contrast, the legacy CareLink 2090 utilizes far‐field RF telemetry in the protected 402–405 MHz MedRadio band, a more robust and interference‐resistant communication method (Table 2).

Furthermore, the SmartSync system's reliance on Bluetooth for its modular components introduces another layer of susceptibility to interference. The older, hardwired 2090 system avoids this vulnerability. From an engineering perspective, this situation suggests that while newer technologies offer enhanced features, they may compromise robustness if not designed with sufficient shielding or cross‐compatibility for legacy devices. This underscores the importance of rigorous cross‐manufacturer testing and supports the practice of maintaining access to older interrogation systems in specialized centers.

**TABLE 2:** Device specifications and technical comparison.

Feature	Medtronic SmartSync [8, 9, 10]	Medtronic/Vitatron CareLink 2090 [9, 11]
Device telemetry method	Inductive coupling (near‐field)	RF telemetry (far‐field)
Telemetry frequency	175 kHz	∼400–406 MHz (MedRadio Band)
Communication mode	Wireless via bluetooth	Wired integrated system
System architecture	Modular (tablet + base + connector)	Standalone integrated unit
EMI susceptibility	Higher (due to low frequency and wireless)	Lower (robust RF protocols)
Internal communication	Bluetooth (2.4 GHz)	Wired connections
Filtering/Signal processing	Proprietary; optimized for 175 kHz/2.4 GHz.	Proprietary; optimized for ∼400 MHz.
Battery operation	Rechargeable	AC‐powered (with backup battery)
Compatibility with MICRA leadless pacemakers	MC1VR01, MC1AVR1, MC2VR01, MC2AVR1	Only MC1VR01 and MC1AVR1

Abbreviations: EMI, electromagnetic interference; GHz, gigahertz; kHz, kilohertz; MHz, megahertz; RF, radio frequency.

## 4 Conclusion

This report details a real‐world instance of programmer telemetry failure arising from suspected EMI from an ILR during interrogation of a leadless pacemaker. The case emphasizes the need for clinicians to be aware of the vulnerabilities in modern programmers when multiple CIEDs are present. It also validates the continued availability of legacy programmers, such as the Medtronic CareLink 2090, though their compatibility with the newest device generations is limited. Our experience suggests a potential troubleshooting strategy: concurrently engaging the interfering device with its dedicated programmer may mitigate EMI and permit successful communication. Ultimately, this case calls for industry‐wide collaboration to improve compatibility testing and to integrate real‐time EMI diagnostics into future programmer designs.

## Ethics Statement

This study was conducted in accordance with the ethical principles of the Declaration of Helsinki.

## Consent

Written informed consent was obtained from the patient for the publication of this case report and any accompanying images. As a retrospective case report of a single patient, formal ethics committee approval was not required.

## Conflicts of Interest

The authors declare that they have no competing interests or conflicts of interest to disclose in relation to this work.

## Data Availability

The data supporting the findings of this case report are not publicly available due to patient privacy and confidentiality restrictions. Further information is available from the corresponding author upon reasonable request.

## References

[1] F. Duru , P. Lauber , G. Klaus , and R. Candinas , “Hospital Pager Systems May Cause Interference With Pacemaker Telemetry,” Pacing and Clinical Electrophysiology 21, no. 11 Pt 2 (1998): 2353–2359. 9825347 10.1111/j.1540-8159.1998.tb01181.x

[2] M. B. Patel , J. P. Thaker , S. Punnam , and K. Jongnarangsin , “Pacemaker Interference With an iPod,” Heart Rhythm 4, no. 6 (2007): 781–784. 17556203 10.1016/j.hrthm.2007.02.018

[3] D. Wang , Y. Sato , K. Nakamura , et al., “Device–Device Interference Triggered by an Abandoned Pacemaker: A Case Report,” European Heart Journal—Case Reports 8, no. 11 (2020): ytae595. 10.1093/ehjcr/ytae595 PMC11561563 39545152

[4] S. Tomomori , J. Yamaguchi , T. Noda , et al., Impella Device Inhibits Telemetry Communication With Implantable Cardioverter‐Defibrillator: A Case Report (JACC Case Report, 2023): 101014.

[5] C. Wu , M. H. Hsieh , Y. J. Lin , et al., “Smartphone Use and Electromagnetic Interference on Cardiac Implantable Electronic Devices: A Cross‐sectional Study,” Journal of the American College of Cardiology 75, no. 11 (2020): 1410–1420.

[6] S. L. Pinski and R. G. Trohman , “Interference in Implanted Cardiac Devices, Part I,” Pacing and Clinical Electrophysiology 25, no. 9 (2002): 1367–1381. 12380774 10.1046/j.1460-9592.2002.01367.x

[7] D. L. Hayes , P. J. Wang , D. W. Reynolds , et al., “Interference With Cardiac Pacemakers by Cellular Telephones,” New England Journal of Medicine 336, no. 21 (1997): 1473–1479. 9154765 10.1056/NEJM199705223362101

[8] Medtronic , CareLink SmartSync™ Device Manager Technical Manual (Medtronic, Inc., 2017).

[9] U.S. Food and Drug Administration , Premarket Approval P150033: Micra Transcatheter Pacing System – Summary of Safety and Effectiveness Data (FDA, 2016).

[10] Medtronic , Pacemaker Programmer With Telemetric Functions. US Patent 4550370. 1985.

[11] Medtronic , CareLink™ 2090 Programmer Reference Manual (Medtronic, Inc., 2012).

